# Optimal pair of hippocampal CA1 phase response curve and spike-timing-dependent plasticity for hetero-associative memory

**DOI:** 10.1186/1471-2202-14-S1-P9

**Published:** 2013-07-08

**Authors:** Ryota Miyata, Keisuke Ota, Toru Aonishi

**Affiliations:** 1 Interdisciplinary Graduate School of Science, Tokyo Institute of Technology, Kanagawa, 226-8502, Japan; 2 Research Fellow of the Japan Society for the Promotion of Science, Tokyo, Japan; 3 Brain Science Institute, RIKEN, Saitama, 351-0198, Japan

## 

Recently reported experimental findings suggest that the hippocampal CA1 network stores spatio-temporal spike patterns and retrieves temporally reversed [1] and spread-out [2] patterns. In this paper, we explore the idea that the properties of the neural interactions and the synaptic plasticity rule in the CA1 network enable it to function as a hetero-associative memory recalling such reversed and spread-out spike patterns. In line with Lengyel's speculation [3], we derive optimally designed spike-timing-dependent plasticity (STDP) rules that are matched to neural interactions formalized in terms of phase response curves (PRCs) for performing the hetero-associative memory function (see Figure 1). First, we formulate a hetero-associative memory network recalling not only the normal spike patterns, but also the reversed and doubly spread-out patterns as a phase oscillator model consisting of an STDP and a PRC. Next, we analytically derive the mutual information between a stored phase pattern and a network output for evaluating memory retrieval performance. By maximizing an object function given by the mutual information, we search for STDP window functions that are optimal for retrieval of normal and doubly spread-out patterns under the constraint that the PRCs are those of CA1 pyramidal neurons recorded *in vitro *[4].

**Figure 1:**
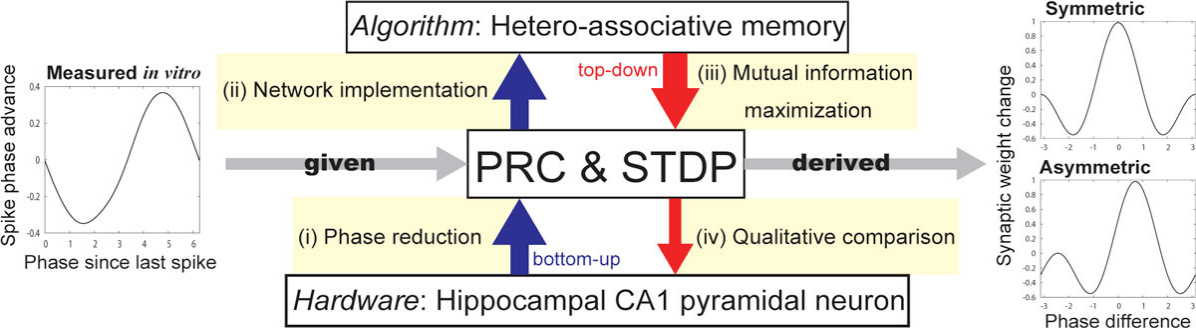
**Outline of our approach to derive pairs of PRCs and STDPs optimally recalling normal, reversed, and doubly spread-out patterns**.

## Conclusions

The typical STDPs observed in CA1 region are classified into two types [5]: symmetric and asymmetric plasticity rules. We show both of these rules are included in the theoretically derived set of optimal STDPs. The theoretically derived STDPs qualitatively coincide with the first two Fourier series approximations for those reported in CA1 neurons. Furthermore, we demonstrate that the system, which can retrieve normal and doubly spread-out patterns, can also retrieve reversed patterns with the same quality.
